# Supplementary material for: Content-rich biological network constructed by mining PubMed abstracts
Source: BMC Bioinformatics. 2004 Oct 8;5:147. doi: 10.1186/1471-2105-5-147 (PMC528731; doi:10.1186/1471-2105-5-147)
Supplement: Additional File 2 — The original results of the above study (non-essential files are deleted to keep the file size under the limit set by BMC bioinformatics). [file 1471-2105-5-147-S2.bz2 › chilibotAdditionalFile2/dip50/713ID8175697E18050/html/SECA_SECB.html]

 


 **SECA** and **SECB** 
  
Found 76 abstracts in PubMed, retrieved 50.  
 

 What does Google say? 
 PDF only 
| .edu only 

---

**Interactive relationship** (e.g. stimulation, inhibition, etc)

**Inhibitory relationship**- This together with the observation that the  **SecA**  mutant protein lacking the C terminal 70 residues had a strongly reduced ability to mediate binding of  **SecB**  precursor complexes to inverted inner membrane vesicles demonstrates that the C terminus of  **SecA**  is also involved in  **SecB**  binding.  Ref: 7713885 J Biol Chem, 1995
- A more than 50 fold difference in affinity for the cytosolic  **SecA**  compared to translocase associated  **SecA**  seems to prevent unproductive binding of  **SecB**  to the cytosolic  **SecA**  and stresses its targeting function.  Ref: 9369228 FEBS Lett, 1997
- Using a chemical cross linking approach, we find that the formation of  **SecA**   **SecB**  complexes is accompanied by a decrease in the level of cross linking of  **SecA**  dimers, suggesting that  **SecB**  induces a conformational change in  **SecA** .  Ref: 11297435 Biochemistry, 2001
- The alkaline phosphatase mutants most sensitive to the loss of  **SecB**  are those most sensitive to inhibition of  **SecA**  via azide treatment, consistent with the necessity for formation of a preprotein  **SecB**   **SecA**  complex.  Ref: 9515905 J Bacteriol, 1998
- **SecB**  and a  **SecB**  precursor complex were shown to inhibit the  **SecA**  mediated lipid vesicle aggregation, suggesting that the overall acidic  **SecB**  protein binds at or near the second lipid binding site on  **SecA** .  Ref: 7713885 J Biol Chem, 1995
- In contrast,  **SecB**  is utilized by only a subset of preproteins to prevent their premature folding and chaperone them to membrane bound  **SecA** .  Ref: 11297435 Biochemistry, 2001
- The preprotein binding molecular chaperone  **SecB**  functions by preventing the premature folding of the preprotein in the cytosol, and targeting it to the peripheral subunit  **SecA**  of the translocase at the cytoplasmic membrane.  Ref: 9369228 FEBS Lett, 1997
**Stimulatory relationship**- Evidence that  **SecB**  enhances the activity of  **SecA** .  Ref: 11297435 Biochemistry, 2001
- **SecB**  is also shown to directly enhance the ATPase activity of  **SecA**  in a concentration dependent and saturable manner.  Ref: 11297435 Biochemistry, 2001
- Biochemical studies suggest that  **SecB**  facilitates precursor targeting by binding to the  **SecA**  protein, a component of the membrane embedded translocation apparatus.  Ref: 10322000 J Bacteriol, 1999
- Ffh, as well as  **SecB**  in Escherichia coli, enhances the binding of  **SecA**  to presecretory proteins in B. subtilis cells.  Ref: 9880811 J Biochem Tokyo , 1999
- The results suggest that  **SecB**  directly enhances the activity of  **SecA**  and provide a biochemical basis for the enhanced transport efficiency of preproteins in the presence of  **SecB**  in vivo.  Ref: 11297435 Biochemistry, 2001
- Treatment of  **SecA**  with a zinc chelator resulted in a loss of the stimulatory effect of  **SecB**  on the  **SecA**  translocation ATPase activity, while the activity could be restored by the addition of ZnCl2.  Ref: 10213615 Biochemistry, 1999
- The equilibrium dialysis experiments and kinetic data support the observation that the  **SecB**  enhanced  **SecA**  ATPase activity is the result of an increased rate of ATP hydrolysis rather than an increase in the affinity of ATP for  **SecA** .  Ref: 11955083 Biochemistry, 2002
**Neutral relationship**- Interaction of  **SecB**  with soluble  **SecA** .  Ref: 9369228 FEBS Lett, 1997
- **SecA**  is thought to recognize  **SecB**  via its carboxy terminus.  Ref: 9321390 EMBO J, 1997
- The molecular chaperone  **SecB**  targets preproteins to  **SecA**  at the translocation sites in the cytoplasmic membrane of Escherichia coli.  Ref: 10213615 Biochemistry, 1999
- The stimulatory effect of preproteins on the interaction between  **SecB**  and  **SecA**  exclusively involves the signal sequence domain of the preprotein, as it can be mimicked by a synthetic signal peptide and is not observed with a mutant preprotein delta8proOmpA bearing a non functional signal sequence.  Ref: 9767586 Mol Microbiol, 1998
- Translocation of these nascent pOmpA chains into inside out membrane vesicles of E. coli required  **SecA**  and depending on size  **SecB** .  Ref: 9593737 J Biol Chem, 1998
- **SecB**  targets the pre protein to  **SecA**  that mediates pre protein translocation through the SecYEG translocon.  Ref: 9564033 EMBO J, 1998
- **SecA**  recognizes  **SecB**  via its carboxyl terminal 22 aminoacyl residues, a highly conserved domain that contains 3 cysteines and 1 histidine residue that could potentially be involved in the coordination of a metal ion.  Ref: 10213615 Biochemistry, 1999
- The C terminus of  **SecA**  is involved in both lipid binding and  **SecB**  binding.  Ref: 7713885 J Biol Chem, 1995
- Preproteins are bound by the cytosolic molecular chaperone  **SecB**  and targeted in a complex with  **SecA**  to the translocation site at the cytoplasmic membrane.  Ref: 8639021 Arch Microbiol, 1996
- The molecular chaperone  **SecB**  is released from the carboxy terminus of  **SecA**  during initiation of precursor protein translocation.  Ref: 9321390 EMBO J, 1997
- The variant forms of  **SecB**  were all capable of interacting with  **SecA**  in solution to form complexes with stability similar to that of complexes between  **SecA**  and wild type  **SecB** .  Ref: 10807917 J Biol Chem, 2000
- A strikingly short amino acid sequence corresponding to only the most distal 22 aminoacyl residues of  **SecA**  suffices for the authentic binding of  **SecB**  or the  **SecB**  precursor protein complex.  Ref: 9321390 EMBO J, 1997
- To determine the minimal requirement for a  **SecB**  binding site, fusion proteins were created between glutathione S transferase and different parts of the carboxy terminus of  **SecA**  and analysed for  **SecB**  binding.  Ref: 9321390 EMBO J, 1997
- Therefore, deletion of the mobile region of  **SecB**  may alter the interactions of  **SecB**  with  **SecA** .  Ref: 10544036 Biochem Biophys Res Commun, 1999
- These data demonstrate that efficient targeting of preproteins by  **SecB**  requires both a functional signal sequence and a  **SecB**  binding domain on  **SecA** .  Ref: 9767586 Mol Microbiol, 1998
- Interaction of  **SecB**  with the  **SecB**  binding domain of  **SecA**  is disrupted by chelators of divalent cations, and could be restored by the addition of Cu2 or Zn2.  Ref: 10213615 Biochemistry, 1999
- In Escherichia coli, the formation of  **SecA**   **SecB**  complexes has a direct effect on  **SecA**  ATPase activity.  Ref: 11955083 Biochemistry, 2002
- The RBP MBP116 hybrid remained  **SecB**  independent and only exhibited a partial export defect in  **secA**  mutant cells.  Ref: 8226642 J Bacteriol, 1993
- We present evidence that at least two regions of  **SecA**  are involved in the formation of active complexes with  **SecB** .  Ref: 10807917 J Biol Chem, 2000
- It is concluded that the  **SecB**   **SecA**  interaction is needed to dissociate the mature preprotein domain from  **SecB** .  Ref: 9767586 Mol Microbiol, 1998
- The C terminal 70 amino acid residues of  **SecA**  are important for efficient in vitro translocation of the  **SecB**  dependent precursor of PhoE across inverted inner membrane vesicles.  Ref: 7713885 J Biol Chem, 1995
- **SecB**  uses its entire molecular surface for these two functions, but for preprotein release and its own membrane release,  **SecB**  relies on the catalytic activity of  **SecA** .  Ref: 11336818 Trends Microbiol, 2001
- In this latter role  **SecB**  demonstrates specific binding to a unique partner,  **SecA** .  Ref: 12475171 Cell Mol Life Sci, 2002
- These findings lend additional support to the proposed pathway for precursor targeting in which  **SecB**  promotes targeting to the translocation apparatus by binding to the  **SecA**  protein.  Ref: 10322000 J Bacteriol, 1999
- This hybrid was dependent upon  **SecB**  for its efficient export and exhibited a strong export defect in  **secA**  mutant cells.  Ref: 8226642 J Bacteriol, 1993
- Heterodimers of wild type  **SecA**  and SecAN880 are defective in  **SecB**  binding, demonstrating that both carboxy termini of the  **SecA**  dimer are needed to form a genuine  **SecB**  binding site.  Ref: 9321390 EMBO J, 1997
- **SecB**  binds to the extreme carboxy terminus of the  **SecA**  ATPase translocase subunit, and this interaction is promoted by preproteins.  Ref: 9767586 Mol Microbiol, 1998
- The crystal structure of the Haemophilus influenzae  **SecB**  provides new insights into how  **SecB**  simultaneously recognizes its two ligands unfolded preproteins and  **SecA** , the ATPase subunit of the translocase.  Ref: 11336818 Trends Microbiol, 2001
- It is concluded that the  **SecB**  binding site on  **SecA**  is confined to the extreme carboxy terminus of the  **SecA**  dimer,.  Ref: 9321390 EMBO J, 1997
- It is concluded that the  **SecB**  binding domain of  **SecA**  is stabilized by a zinc ion that promotes the functional binding of  **SecB**  to  **SecA** .  Ref: 10213615 Biochemistry, 1999
- The chaperone  **SecB**  keeps precursor proteins in a translocation competent state and targets them to  **SecA**  at the translocation sites in the cytoplasmic membrane of Escherichia coli.  Ref: 9321390 EMBO J, 1997
- sHsps,  **SecB**  the energy dependent step is performed by another chaperone Hsp70,  **SecA** .  Ref: 9563819 Biol Chem, 1998
- To gain more insight into the functional interaction of  **SecB**  and  **SecA** , in vivo, mutations in the  **secA**  locus that compensate for the export defect caused by the  **secB**  missense mutation secBL75Q were isolated.  Ref: 10322000 J Bacteriol, 1999
- The Escherichia coli secretion dedicated chaperone  **SecB**  targets a subset of proteins to the translocase by interacting with the carboxyl C terminus of  **SecA** .  Ref: 11021932 Microbiology, 2000
- It appears that  **SecB**  may function as an intermolecular regulator of ATP hydrolysis by promoting the ATP bound state of  **SecA** .  Ref: 11955083 Biochemistry, 2002
- **SecA**  ATPase promotes Escherichia coli protein translocation by its association with the preprotein or preprotein  **SecB**  complex, anionic phospholipids, and the other core component of translocase, integral membrane protein SecYEG.  Ref: 9111035 J Biol Chem, 1997
- During localization to the periplasmic space or to the outer membrane of Escherichia coli some proteins are dependent on binding to the cytosolic chaperone  **SecB** , which in turn is targeted to the membrane by specific interaction with  **SecA** , a peripheral component of the translocase.  Ref: 10807917 J Biol Chem, 2000
- **SecB**  binds to a subset of precursor proteins, and targets them in a translocation competent state to the  **SecA**  subunit of the translocase.  Ref: 7543278 Biochemistry, 1995

**Non-interactive relationship** (e.g. studied together, co-existance, homology, etc.)

- The most active preparations obtained upon purification were those containing a soluble  **SecA**   **SecB**  complex.  Ref: 8175697 J Biol Chem, 1994
- Overproduction of  **SecA**  suppresses the export defect caused by a mutation in the gene encoding the Escherichia coli export chaperone  **secB** .  Ref: 10322000 J Bacteriol, 1999
- Regions of maltose binding protein that influence  **SecB**  dependent and  **SecA**  dependent export in Escherichia coli.  Ref: 8226642 J Bacteriol, 1993
- These data suggest that SRP FtsY and  **SecB**   **SecA**  constitute distinct targeting routes.  Ref: 10514469 J Biol Chem, 1999
- ... secretory proteins are directed into the  **SecA**   **SecB**  mediated post translational targeting pathway by means of their preferential recognition by trigger factor.  Ref: 10619852 EMBO J, 2000
- Inhibition of  **SecA**  with sodium azide and mutations in  **SecB** , SecD, and SecF, all of which slow down protein secretion, result in biotinylation of PSBT.  Ref: 8655479 J Bacteriol, 1996
- Recent evidence suggests that in Escherichia coli,  **SecA**   **SecB**  and signal recognition particle SRP are constituents of two different pathways targeting secretory and inner membrane proteins to the SecYEG translocon of the plasma membrane.  Ref: 10931878 J Cell Biol, 2000
- Export after  **SecA**  depletion was observed in cells that lack  **SecB** , the primary targeting factor for OMPs, but was abolished by partial inactivation of DnaK.  Ref: 12403776 J Biol Chem, 2002
- Besides  **SecA**  and  **SecB** , Escherichia coli cells possess a signal recognition particle SRP to target exported proteins to the SecY translocon.  Ref: 10619852 EMBO J, 2000
- The characterization of a spontaneous TorA signal peptide mutant TorA\*, in which the two arginine residues in the c region had been replaced by one leucine residue, showed that the TorA\* MalE mutant precursor had acquired the ability for efficiently using the  **SecB**   **SecA**  pathway.  Ref: 12700260 J Bacteriol, 2003
- When preLamB was synthesized in the presence of this material, a considerable amount of precursor was recovered from a soluble ternary complex consisting of preLamB,  **SecA** , and  **SecB** .  Ref: 8175697 J Biol Chem, 1994
- The most intriguing observation is that translocation of the TMAO reductase across the cytoplasmic membrane is independent of the SecY, SecE,  **SecA**  and  **SecB**  proteins.  Ref: 9427745 EMBO J, 1998
- Furthermore, functional signal peptides, but not dysfunctional ones, promote the formation of  **SecA**   **SecB**  complexes.  Ref: 11297435 Biochemistry, 2001
- The proteolysis data indicate that  **SecB**  and the nonhydrolyzable ATP analogue AMP P C P induce similar conformational changes in  **SecA**  which result in a more open or extended structure that is suggestive of the ATP bound form.  Ref: 11955083 Biochemistry, 2002
- Identification of a soluble  **SecA**   **SecB**  complex by means of a subfractionated cell free export system.  Ref: 8175697 J Biol Chem, 1994
- However,  **SecA**  and  **SecB**  cotranslationally associated with nascent pOmpA, since they could be co isolated with the ribosome associated nascent chains and as such catalyzed translocation subsequent to the release of the ribosome.  Ref: 9593737 J Biol Chem, 1998
- In vitro studies with purified components reveal signal recognition particle SRP and  **SecA**   **SecB**  as constituents of two independent protein targeting pathways of Escherichia coli.  Ref: 10397756 Mol Biol Cell, 1999
- The incorporation of sequence motifs in the mature region, which confer  **SecB**  dependence in vivo, had no impact on  **SecA**  activation in vitro.  Ref: 11969418 Biochemistry, 2002
- This was achieved in a novel cell free system from E. coli which, by extensive subfractionation, was simultaneously rendered deficient in  **SecA**   **SecB**  and the signal recognition particle SRP components, Ffh P4.  Ref: 10397756 Mol Biol Cell, 1999
- The inhibition of  **SecA**  ATPase activity by sodium azide in the presence of IMVs and a functional signal peptide further indicates that  **SecB**  promotes the ATP bound form of  **SecA** .  Ref: 11955083 Biochemistry, 2002
- These results indicate that SRP dependent integration and  **SecA**   **SecB**  mediated translocation do not only represent two independent protein delivery systems, but also remain mechanistically distinct processes even at the level of the membrane where they engage different domains of SecY and different components of the translocon.  Ref: 10931878 J Cell Biol, 2000
- In concert with  **SecB** ,  **SecA**  targets the precursors to SecY and energizes their translocation by its ATPase activity.  Ref: 11051763 Prog Nucleic Acid Res Mol Biol, 2001
- PreLamB synthesized in this manner is translocated into salt extracted plasma membrane vesicles only in the presence of  **SecA**   **SecB**  or  **SecB**  containing preparations of the E. coli cytosol.  Ref: 8175697 J Biol Chem, 1994
- In addition, an accumulation of pre MglB in  **secB** ,  **secA**  and secY mutants suggested that  **SecB**  and the Sec translocase are also involved in export of the periplasmic galactose receptor.  Ref: 8828228 Microbiology, 1996
- Furthermore, they suggest that SRP and  **SecA**   **SecB**  have different substrate specificities resulting in two separate targeting mechanisms for membrane and secretory proteins in E. coli.  Ref: 10397756 Mol Biol Cell, 1999
- An artificial transmembrane segment directs  **SecA** ,  **SecB** , and electrochemical potential dependent translocation of a long amino terminal tail.  Ref: 10037778 J Biol Chem, 1999
- Precursor specific requirements for  **SecA** ,  **SecB** , and delta muH during protein export of Escherichia coli.  Ref: 8175698 J Biol Chem, 1994
- The integration of two membrane proteins into inside out plasma membrane vesicles of E. coli required all three SRP components and could not be driven by  **SecA** ,  **SecB** , and DeltamicroH.  Ref: 10397756 Mol Biol Cell, 1999
- In Escherichia coli, the  **SecB**   **SecA**  branch of the Sec pathway and the twin arginine translocation Tat pathway represent two alternative possibilities for posttranslational translocation of proteins across the cytoplasmic membrane.  Ref: 12700260 J Bacteriol, 2003
- The nature of the interaction of  **SecB**  with soluble  **SecA**  was studied by fluorescence anisotropy spectroscopy of Ru bpy 2 dcbpy labeled  **SecA**  in the presence of increasing concentrations of  **SecB** .  Ref: 9369228 FEBS Lett, 1997
- **SecB**  reduces the translocation of delta8proOmpA into these vesicles and almost completely prevents translocation when, in addition, the  **SecB**  binding site on  **SecA**  is removed.  Ref: 9767586 Mol Microbiol, 1998
- In the presence of high concentrations of both  **SecB**  and  **SecA** , the precursors were translocated in vitro with indistinguishable kinetics.  Ref: 9484900 Mol Microbiol, 1998
- **SecB**  modulates the nucleotide bound state of  **SecA**  and stimulates ATPase activity.  Ref: 11955083 Biochemistry, 2002
- Discrimination between SRP and  **SecA**   **SecB**  dependent substrates involves selective recognition of nascent chains by SRP and trigger factor.  Ref: 10619852 EMBO J, 2000
- This region of  **SecA**  is highly conserved in Eubacteria, but despite its presence in the Bacillus subtilis  **SecA** , the B. subtilis genome does not appear to contain a gene for a clear homologue of  **SecB** .  Ref: 11021932 Microbiology, 2000
- Complexes between protein export chaperone  **SecB**  and  **SecA** .  Ref: 10807917 J Biol Chem, 2000
- Complex formation between  **SecA**  and  **SecB**  was verified by co sedimentation and co immunoprecipitation.  Ref: 8175697 J Biol Chem, 1994
- The accumulation of secretory protein precursors, caused either by mutations in  **secB**  or  **secA**  or by the overproduction of export defective proteins, results in a two to fivefold increase in the synthesis of heat shock proteins.  Ref: 8320215 J Bacteriol, 1993
- Using purified components and synthetic signal peptides, we have studied the interaction of  **SecB**  with  **SecA**  and with  **SecA**  signal peptide complexes in vitro.  Ref: 11297435 Biochemistry, 2001
- **SecA**  and  **SecB** , although required for translocation, did not mediate binding of the ribosome associated pOmpA to membrane vesicles.  Ref: 9593737 J Biol Chem, 1998
- The structural complexity of a ligand in association with the molecular chaperones  **SecB**  and  **SecA**  was investigated using three species of precursor maltose binding protein, which differ in their stability as a result of an amino acid substitution in each that affects the rate of folding of the polypeptide.  Ref: 9484900 Mol Microbiol, 1998
- In Escherichia coli, three different types of proteins engage the SecY translocon of the inner bacterial membrane for translocation or insertion 1 polytopic membrane proteins which prior to their insertion into the membrane are targeted to the translocon using the bacterial signal recognition particle SRP and its receptor, 2 secretory proteins which are targeted to and translocated across the SecY translocon in a  **SecA**  and  **SecB**  dependent reaction and 3 membrane proteins with large periplasmic domains, requiring SRP for targeting and  **SecA**  for the translocation of the periplasmic moiety.  Ref: 12682042 J Biol Chem< MedlineTA>J Biol Chem, 1998
- Our results suggest that a soluble  **SecA**   **SecB**  complex participates in the export of preLamB.  Ref: 8175697 J Biol Chem, 1994
- Zinc stabilizes the  **SecB**  binding site of  **SecA** .  Ref: 10213615 Biochemistry, 1999
- **SecA**  dependent binding of E. coli  **SecB**  to membrane vesicles was observed when E. coli  **SecA**  was used, but not with the S. lividans  **SecA** , suggesting that this interaction may be specific for the Gram negative bacteria.  Ref: 9826195 Eur J Biochem, 1998
- To determine the biological consequence of this finding, the influence of  **SecB**  on the signal peptide stimulated  **SecA**  lipid ATPase was studied using synthetic peptides of varying hydrophobicity.  Ref: 11297435 Biochemistry, 2001
- We compare translocation into inside out plasma membrane vesicles INV of the in vitro synthesized outer membrane proteins LamB and OmpA and the periplasmic protein Skp of Escherichia coli and demonstrate a precursor specific dependence on the export factors  **SecA** ,  **SecB** , and the proton motive force delta mu H.  Ref: 8175698 J Biol Chem, 1994
- Discrimination between  **SecA**   **SecB**  and SRP dependent targeting involves the specificity of SRP for hydrophobic signal anchor sequences and the exclusion of SRP from nascent chains of translocated proteins by trigger factor, a ribosome associated chaperone.  Ref: 11051763 Prog Nucleic Acid Res Mol Biol, 2001
- In contrast, here we describe alkaline phosphatase mutants containing artificial transmembrane segments that demonstrate that translocation of a long N tail across the membrane is dependent upon  **SecA** ,  **SecB** , and the electrochemical potential in the absence of a signal peptide.  Ref: 10037778 J Biol Chem, 1999
- Preprotein transfer to the Escherichia coli translocase requires the co operative binding of  **SecB**  and the signal sequence to  **SecA** .  Ref: 9767586 Mol Microbiol, 1998
- **SecB**  is released from the translocase at a very early stage in protein translocation when the membrane bound  **SecA**  binds ATP to initiate translocation.  Ref: 9321390 EMBO J, 1997
